# Dissipative Particle Dynamics Simulations for Shape Change of Growing Lipid Bilayer Vesicles

**DOI:** 10.3390/life13020306

**Published:** 2023-01-22

**Authors:** Hiromi Mitsuhashi, Ryota Morikawa, Yoh Noguchi, Masako Takasu

**Affiliations:** School of Life Sciences, Tokyo University of Pharmacy and Life Sciences, Tokyo 192-0392, Japan

**Keywords:** vesicle, lipid, DPD method, growing membrane, L-form cell, tubulation, budding

## Abstract

The characteristic shape changes observed in the growth and division of L-form cells have been explained by several theoretical studies and simulations using a vesicle model in which the membrane area increases with time. In those theoretical studies, characteristic shapes such as tubulation and budding were reproduced in a non-equilibrium state, but it was not possible to incorporate deformations that would change the topology of the membrane. We constructed a vesicle model in which the area of the membrane increases using coarse-grained particles and analyzed the changes in the shape of growing membrane by the dissipative particle dynamics (DPD) method. In the simulation, lipid molecules were added to the lipid membrane at regular time intervals to increase the surface area of the lipid membrane. As a result, it was found that the vesicle deformed into a tubular shape or a budding shape depending on the conditions for adding lipid molecules. This suggests that the difference in the place where new lipid molecules are incorporated into the cell membrane during the growth of L-form cells causes the difference in the transformation pathway of L-form cells.

## 1. Introduction

**Figure 1:**
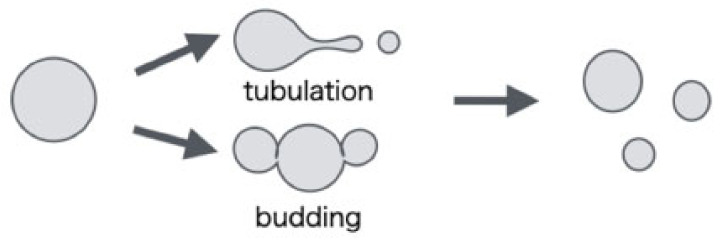
Schematic diagram of the major cell division shapes of L-form cell.

Existing cells use a complex mechanism in order to grow and divide. Primitive cells, on the other hand, do not have such a complex mechanism and can undergo protein-free cell division. Therefore, biochemical and simulation studies have been conducted to investigate primitive cell division. One of the primitive cell models used in biochemical experiments is L-form cells with gene mutations that inhibit cell wall formation [1,2]. L-form cells have been observed in various bacteria such as *Bacillus subtilis*, *Escherichia coli*, and *Listeria monocytogenes* [3,4,5]. Irregular shape changes are observed in the cell division of L-form cells as shown in the Figure 1.

Mercier et al. used *B*. *subtilis* to study the characteristic division mechanism found in this L-form cells [6]. In their study, experiments were conducted using a wild-type strain of *B. subtilis* and a protoplast of its mutant strain. The protoplasts are cells from which the cell walls of plant cells and bacteria have been removed by enzymatic treatment, and have a spherical shape under isotonic pressure. However, while protoplasts from wild-type do not cause cell division, the protoplasts from mutant with gene mutations which overproduce cell membranes were found to undergo irregular cell division like L-form cell. Furthermore, it was also revealed that by removing the cell wall after growing a wild-type strain that inhibited cell division, the wild-type strain divides like L-form cells. What all these experimental results have in common is that when the cell walls of rod cells and spherical cells are removed to make spherical cells, extra membranes are formed on these cells and irregular division occurs. Based on this result, the cell division mechanism could be explained by physical factors based on elasticity theory of the cell membrane [7]. That is, the excessive growth of the cell membrane relative to the cytoplasm creates an excess membrane region that allows cell deformation, and cell division occurs.

In order to analyze the deformation and division of L-form cells quantitatively, it is a very meaningful modeling to regard L-form cells as vesicles, which are closed lipid bilayer membranes. Ziherl et al. theoretically investigated the stable shape of symmetric vesicles based on the bending elasticity model of the membrane [8]. Since they performed the analysis using the area differential elasticity (ADE) model, it was not possible to consider the topological deformation of the membrane [9]. Grafümuler et al. also analyzed how vesicles fuse with lipid bilayer membranes [10]. They composed coarse-grained particles of water and lipid molecules and simulated them using the dissipative particle dynamics (DPD) method [11]. As a result, it was clarified that the tension of the lipid bilayer needs to exceed a certain threshold for the fusion of the lipid bilayer and the vesicle.

Baoukina et al. performed simulation analysis of the topological changes in a single layer membrane, such as the formation of bucklings or semivesicles, which are observed when pressure is applied to both ends of the single layer membrane formed at the interface between water and air [12]. They performed MD calculations using coarse-grained lipid molecules and found that the topological changes of the membrane were controlled by macroscopic parameters such as curvature elasticity and tension of the membrane. Furthermore, Nakagawa et al. found that the formation of bilayer sheet protrusions (BP) in bilayers and vesicles is induced by hydrolysis and condensation reactions of amphipathic molecules [13]. It was clarified that the relative ratio of the viscosity in the bilayer membrane and the surrounding viscosity is important for the formation of BP, and that the higher the viscosity of the surrounding fluid, the more BP is formed. They also found that vesicle division was observed by hydrolysis and condensation reaction of lipids.

Ruiz-Herrero et al. used a simulation of the triangular lattice membrane model to investigate the shape change in the non-equilibrium state as observed during the division of L-form cells [14]. They simulated using a growth vesicle model that took into account the growth of lipid membranes and the penetration of water in the membranes. As a result, when the value of the dimensionless parameter indicating the magnitude of the relative spontaneous curvature is positive, the shape (tubulation, budding) that forms small vesicles on the outside is observed, and when the value is negative, a shape that forms small vesicles inside (vesiculation) is obtained. On the other hand, when the dimensionless parameter indicating the strength to grow beyond the critical radius is large, the amount of increase in both the volume and surface area of the vesicle becomes faster than the relaxation rate, so that elastic energy accumulates in the membrane. Small vesicles were formed on the entire surface of the vesicle so as to relieve the elastic energy. However, since the model did not allow changes in topology, the shape of the vesicles observed was limited. In addition, as revealed by Ziherl et al. [8], the difference between the area of the outer membrane surface and the area of the inner membrane surface of the vesicle plays an important role in determining the shape. On the other hand, Ruiz-Herrero’s model is composed of a single layer of membrane, and the area difference of the membranes is not considered. Therefore, the above-mentioned problem can be avoided by allowing the topological change and performing the simulation using the model that accurately represents the lipid bilayer membrane.

In this paper, we constructed a model of a vesicle with an increasing membrane area and simulated it in order to investigate the reason why L-shaped cells may reach division by different deformation pathways, as shown in Figure 1. Although cell division in bacteria requires the prokaryotic cytoskeletal protein FtsZ, it is known that L-form cells without cell walls undergo characteristic cell division independent of FtsZ. Therefore, in order to understand the mechanism of cell division in L-form cells or primitive cells without cell walls, it is important to investigate the division mechanism of vesicles composed only of lipid molecules and the deformation during the process from a physical point of view. Analysis using molecular simulation, in particular, is very meaningful because it can show the correlation between the deformation of the vesicle and the behavior of the lipid molecules that make up the vesicle. Specifically, we investigate the characteristics of the shape change of the growing vesicles in the non-equilibrium state by using a model in which the molecule is coarse-grained by the dissipative particle dynamics (DPD) method [11]. In this model, not only the topological changes of vesicle are allowed, but also lipid molecules are added to the system at regular time intervals in order to increase the surface area of the lipid bilayer membrane. Since the number of particles in the system increases with time, the simulation is performed under a constant pressure, and the size of the system changes accordingly.

We find that the vesicle deformed into a tube shape when lipid molecules were added uniformly to the lipid bilayer. On the other hand, when a lipid molecule is added only to one layer of the lipid bilayer, semi-vesicle-like structures are generated inside or outside the vesicle through topological changes. Since the result of the former condition is a deformation corresponding to “tubulation” and the latter condition is the deformations corresponding to “budding”, the molecular transport process by which lipid molecules are incorporated into the lipid bilayer is important and might be involved in the path selection of these deformations in L-form cells.

## 2. Models and Methods

### 2.1. Forces Acting between DPD Particles

**Figure 2:**
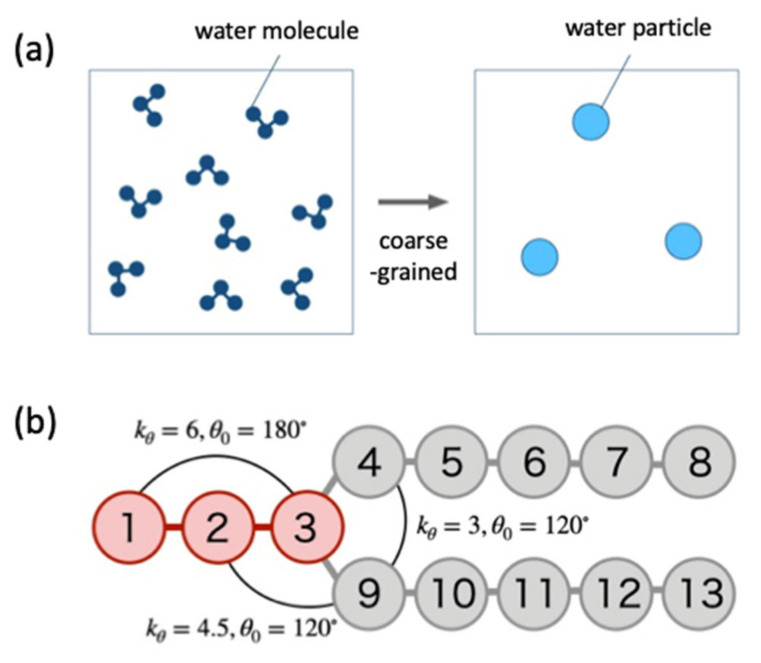
Coarse graining of water and lipid molecules using DPD particles. (**a**) The three water molecules are combined into one and are considered as one water particle (light blue). (**b**) Lipid molecule is composed of hydrophilic part (red) and hydrophobic part (gray). The parameters related to the angular potential are set at three different values in the lipid molecule.

A vesicle was constructed in water using coarse-grained water and lipid molecules, and we simulated this model using the Dissipative Particle Dynamics (DPD) [11,15]. The DPD is a method devised to calculate a fluid, and the fluid is represented by soft particles which move according to a collision law. These coarse-grained particles are called DPD particles. In addition, by binding DPD particles to each other with a spring to give elasticity, it is possible to simulate not only fluids but also polymers such as lipid membranes [16]. In this paper, as our model shown in Figure 2, three water molecules are grouped together and represented by one DPD particle, and one lipid molecule is represented by 13 DPD particles [17].

The external force $F_{i}$ applied to a water particle i is the sum of the conservative force $F_{ij}^{C}$, the dissipative force $F_{ij}^{D}$, and the random force $F_{ij}^{R}$ from the neighboring unbonded particle j, and is expressed by the following formula [18]:

(1) $$\begin{array}{cc} F_{i} & =\sum_{i\neq j}\left(F_{ij}^{C}+F_{ij}^{D}+F_{ij}^{R}\right)\\ & =\sum_{i\neq j}\left\{a_{ij}\omega \left(r_{ij}\right){\hat{r}}_{ij}-\gamma \omega^{2}\left(r_{ij}\right)\left({\hat{r}}_{ij}\cdot v_{ij}\right){\hat{r}}_{ij}+\sigma \omega \left(r_{ij}\right)\xi_{ij}\Delta t^{-1/2}{\hat{r}}_{ij}\right\}. \end{array}$$

Here, $a_{ij}$ is the maximum repulsive force between particles i and j, and the values used in the simulation are shown in Table 1 [19]. $r_{ij}=r_{ij}\;{\hat{r}}_{ij}$ is the distance between particles i and j, where ${\hat{r}}_{ij}$ is the unit vector of $r_{ij}$. $r_{ij}$ is the relative velocity between particles i and j. Δt is the time step in the simulation, which is also a unit time in the model. The sum of the forces of the particles i and j is calculated within its cutoff length $r_{c}$ which is treated as a unit length. The parameter γ corresponds to the strength of the dissipation, and the parameter $\sigma =\sqrt{2\gamma k_{B}T}$ is the strength of the noise with the temperature $k_{B}T$. $\xi_{ij}$ is a random number with variance 1 and average 0 and $\omega \left(r_{ij}\right)=1-r_{ij}/r_{c}$ ($r_{ij}<r_{c}$) is $r_{ij}$-dependent weight function [20].

In addition to the force $F_{i}$, the elastic harmony force $F_{ij}^{S}$ and the force $F^{\theta }$ due to the angular potential act between the bound particles that make up the lipid molecule:

(2) $$F_{ij}^{S}=k_{s}\left(1-r_{ij}/r_{s}\right){\hat{r}}_{ij},$$

(3) $$F^{\theta }=-\nabla \left\{k_{\theta }{\left(\cos\theta -\cos\theta_{0}\right)}^{2}\right\}.$$

Here, $k_{s}$ is the spring constant between particles which make up lipid, and $r_{s}$ is the natural length of the spring. In the simulation, we set $k_{s}=100$ and $r_{s}=0.7r_{c}$. On the other hand, $k_{\theta }$ is the bending constant, θ is the angle between the two bonds and $\theta_{0}$ is the natural angle. In the lipid molecule used in the simulation, the force $F^{\theta }$ depending on the bond angle is set for the three points in the lipid molecule as shown in Figure 2b, and the values of $k_{\theta }$ and $\theta_{0}$ are different.

### 2.2. Construction of Vesicles and Addition of Lipid Molecules under Constant Pressure

In order to mimic the shape of growing L-form cell, a simulation is performed using a vesicle consisting of DPD particles of water and lipid. The system to be simulated is a cubic box with a size of $50r_{c}\times 50r_{c}\times 50r_{c}$ and has periodic boundary conditions. The simulation box is initially filled with 375,000 DPD particles so that the system density is ρ=3, and a vesicle and water particles are placed in the box. The vesicle is formed by assembling lipid molecules as a single closed bilayer membrane.

**Figure 3:**
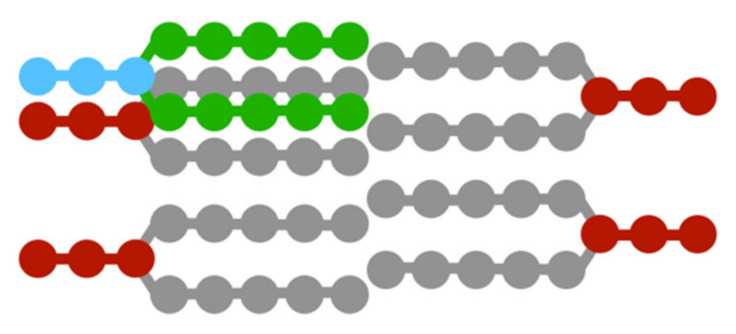
Addition of lipid molecule. The added lipid is shown in light blue and green.

In the simulation, lipid molecules are added into the lipid bilayer over time in order to simulate shape changes during the growth of L-form cells. Lipid molecules, which are the materials for growing cell membranes, are biologically biosynthesized in the endoplasmic reticulum and Golgi apparatus and transported to the desired location. However, in order to reduce the simulation time, the lipid molecules are placed directly in the lipid bilayer membrane. Since the DPD method uses a soft potential that allows the overlapping of particles, the particles can be placed at any coordinate. Therefore, as a method of adding lipid molecules, first, one lipid molecule in the lipid bilayer is randomly selected and duplicated. Then, by arranging the duplicated lipid molecule slightly offset from the original lipid molecule, the lipid molecule is added in the lipid bilayer membrane (Figure 3).

By adding lipid molecules, the number of DPD particles in the simulation box increases and the pressure of the system also increases. Therefore, in order to perform the simulation under natural conditions, it is necessary to perform the simulation with constant pressure $P_{0}$ in the system. Here, an extended system method proposed by Andersen is used to perform DPD simulations with constant pressure [21]. The extended system is a method of calculating motion with a system that adds a new degree of freedom (barostat) to the degree of freedom of a particle, and can control a macroscopic quantity in the target particle system. In general MD simulation, it is necessary to combine two expanded systems, one with constant pressure and one with constant temperature. in order to control pressure and temperature at the same time. However, in the DPD method, the temperature is conserved by the dissipative force and the random force, so the pressure and temperature can be controlled by adding only an extended system with a constant pressure.

Thus, the DPD simulation is performed by the modified velocity Verlet method with Andersen barostat, which is a typical extended system with constant pressure [22].

### 2.3. Direction Vector of Lipid Molecule and Unit Normal Vector of Membrane Surface

In order to understand the microscopic state of the vesicular membrane, we analyzed the shape and orientation of the individual lipid molecules that make up the vesicle. Therefore, from the simulation results, we estimate the tilt angle $\psi_{k}$ defined between the direction of the lipid molecule k and the normal vector of the bilayer vesicle in the vicinity of lipid molecule k.

First, we define the three direction vectors $u_{\mathrm{phi},k}$, $u_{\mathrm{pho},k}$ and $u_{k}$ in the lipid molecule k which represent the directions of the hydrophilic group, the hydrophobic group, and the entire lipid molecule, respectively. The direction vector $u_{\mathrm{phi},k}$ is defined by the vector from the 3rd DPD molecule to the 1st DPD molecule in Figure 2b. The vector $u_{\mathrm{pho},k}$ representing the direction of the hydrophobic group is defined by the sum of the direction vectors $u_{\mathrm{pho}1,k}$ from the 8th DPD particle to the 3rd DPD particle and $u_{\mathrm{pho}2,k}$ from the 13th DPD particle to the 3rd DPD particle. The direction vector representing the entire lipid molecule k is calculated by $u_{k}=u_{\mathrm{phi},k}+u_{\mathrm{pho},k}$.

Secondly, in order to calculate the normal vector of the bilayer membrane that composes the vesicle, it is necessary to approximate the shape of the thick vesicle to the closed surface $R\left(\theta ,\;\phi \right)$. Here, θ and ϕ are the declinations in the spherical coordinate system with the center of gravity of the vesicle as the origin. When the shape of the vesicle is close to spherical, the vector $R\left(\theta ,\;\phi \right)$ representing the shape of the vesicle is expanded as follows using the spherical harmonics $Y_{l}^{m}\left(\theta ,\phi \right)$ up to the degree l=2:

(4) $$R\left(\theta ,\phi \right)=\overset{2}{\underset{l=0}{\sum }}\overset{+l}{\underset{m=-l}{\sum }}c_{l}^{m}Y_{l}^{m}\left(\theta ,\phi \right).$$

Assuming that the DPD particles are uniformly packed, weights for spherical harmonics $c_{l}^{m}=\left(c_{x,l}^{m},c_{y,l}^{m},c_{z,l}^{m}\right)$ is approximated as follows:

(5) $$c_{l}^{m}=\frac{4\pi {\bar{R}}^{2}}{N_{p}}\overset{N_{p}}{\underset{i=1}{\sum }}\frac{R_{i}}{R_{i}^{2}}Y_{l}^{m}\left(\theta_{i},\phi_{i}\right),$$

where $R_{i}=\left(R_{i}\sin\theta_{i}\cos\phi_{i},\;R_{i}\sin\theta_{i}\cos\phi_{i},\;R_{i}\cos\theta_{i}\right)$ is the coordinate vector of the DPD particle $i\;\left(=1\sim N_{p}\right)$ of the lipid molecule when the center of gravity of the vesicle is the origin, and $\bar{R}=\left(1/N_{p}\right)\sum_{i=1}^{N_{p}}R_{i}$ is the average radius of the vesicle. By using the value of $c_{l}^{m}$ obtained in Equation (5), the normal vector of the bilayer vesicle in the vicinity of lipid molecule k is defined as [23]:

(6) $$n_{k}=\left(\overset{2}{\underset{l=0}{\sum }}\overset{l}{\underset{m=-l}{\sum }}c_{l}^{m}{\left.\frac{\partial Y_{l}^{m}\left(\theta ,\phi \right)}{\partial \theta }\right|}_{\theta =\theta_{k},\phi =\phi_{k}}\right)\times \left(\overset{2}{\underset{l=0}{\sum }}\overset{l}{\underset{m=-l}{\sum }}c_{l}^{m}{\left.\frac{\partial Y_{l}^{m}\left(\theta ,\phi \right)}{\partial \phi }\right|}_{\theta =\theta_{k},\phi =\phi_{k}}\right).$$

Here, $\theta_{k}$ and $\phi_{k}$ are the declinations in the spherical coordinate system that represent the coordinates of the center of gravity of the lipid molecule k. From the above, the tilt angle $\psi_{k}$ of the lipid molecule k with respect to the bilayer vesicle is calculated as follows:

(7) $$\psi_{k}=\cos^{-1}\left(\frac{n_{k}}{\left|n_{k}\right|}\cdot \frac{u_{k}}{\left|u_{k}\right|}\right).$$

When the shape of the vesicle is close to a sphere, the direction vectors of these lipid molecules $u_{k}$ are almost opposite in the inner and outer layers of the lipid bilayer. Therefore, when calculating $\psi_{k}$ in the inner layer of the membrane, the sign of the normal vector of the vesicle $n_{k}$ in Equation (6) is taken in reverse. Using the tilt angle $\psi_{k}$ of lipid molecules in either inner or outer layers of the lipid bilayer, the order parameter S of the director of the lipid molecules is given by:

(8) $$S=\frac{1}{2}\langle 3\cos^{2}\psi_{k}-1\rangle .$$

Here ⟨⋯⟩ represents the average for the lipid molecules in either inner or outer layers of the lipid bilayer.

### 2.4. Clustering Water Particles

As the addition of lipid molecules to the vesicle progresses, the vesicle may undergo topological deformation. To investigate the morphological features of vesicles caused by topology deformation, such as the internal structure of vesicles, we analyze the number and size of groups of water particles separated by the lipid bilayer membranes that make up the vesicles. Here, these groups of water particles are called clusters, and the method of dividing them according to certain rules is called clustering.

**Figure 4:**
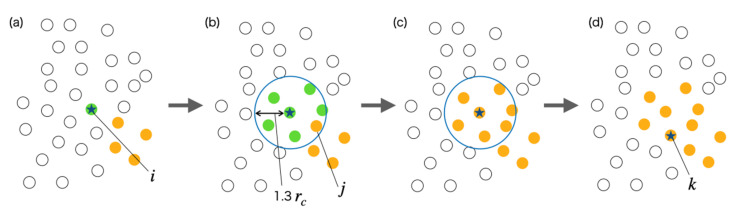
Explanatory diagram of clustering algorithm. (**a**) Particle with star is a standard particle of the cluster, and white particles are unclassified in clusters. (**b**) The particle j within a certain distance of $1.3r_{c}$ from the reference particle i is selected. (**c**) Since particle j has already been classified into another cluster β (orange), all particles classified into cluster α are incorporated into cluster β. (**d**) Particle within a distance of $1.3r_{c}$ from the reference particle i that has not yet been selected is selected as the next reference particle k.

The algorism of clustering is shown in Figure 4 and the procedure is as follows:

(1) First, the reference particle i is selected from the water particles inside the vesicle and classified into cluster α (Figure 4a).
(2) Next, the particles j within a certain distance of $1.3r_{c}$ from the reference particle i are classified into cluster α (Figure 4b). However, if the particle j has already been classified into another cluster β, all the particles classified into cluster α are incorporated into cluster β (Figure 4c).
(3) Particle within a distance of $1.3r_{c}$ from the reference particle i that has not yet been selected as the reference particle is selected as the next reference particle k (Figure 4d).
(4) If the particle k does not exist, the unclassified water particle l is used as the new reference particle.
(5) Repeat steps (1) to (4) until all water particles are classified into some cluster.

We used this clustering method to determine the regions of water molecules separated by the lipid bilayer, and from the number of the clusters, we can know the Euler characteristic of the shape of the vesicle.

## 3. Results

### 3.1. Construction of Initial Structure

In order to construct the initial structure of the vesicle used in the simulation, a spherical vesicle with a radius of $15r_{c}$ consisting of 4000 lipid molecules is first placed in the system together with 323,000 water particles. Here, 27,289 water particles are contained inside the spherical vesicle. Next, while keeping the temperature and pressure of this system constant, the simulation is performed for 100,000 steps with the time step Δt=0.01 and the parameter γ=4.5, and the system is sufficiently relaxed. The pressure and temperature of the system are set to $P_{0}=23.649$ and $k_{B}T=1$ as in the simulation of adding lipids.

**Figure 5:**
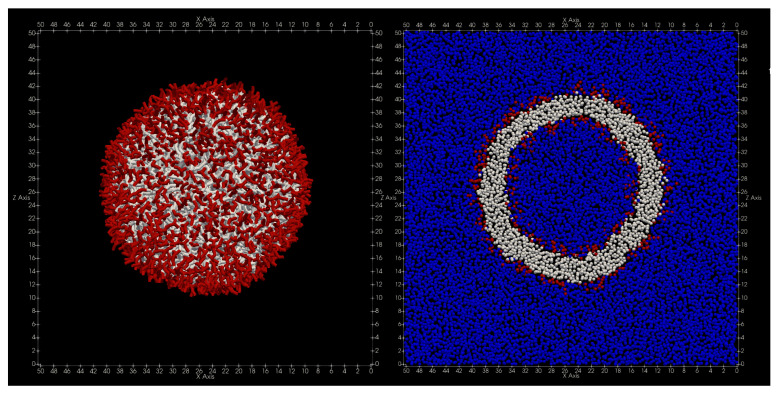
The shape of the vesicle relaxed under constant pressure and constant temperature. Blue is water particles, red is hydrophilic group particles, and white is sparse water group particles. (Left figure: appearance, right figure: cross section).

The shape of the finally obtained vesicle is shown in Figure 5. Immediately after the start of the simulation, the pressure value became constant immediately, and the volume, temperature, and energy values became constant after 20,000 steps. Additionally, the radius of inertia of the vesicles became nearly constant around $R_{g}=11.9r_{c}$ after 50,000 steps.

During this relaxation process, water particles inside and outside the vesicle did not penetrate the lipid bilayer membrane. Therefore, if we investigate whether the water particles closest to the lipid molecule are inside or outside the vesicle, we could distinguish whether the lipid molecules that make up the vesicle are in the inner layer or the outer layer of the membrane. However, if the vesicle undergoes a large deformation and its topology changes, it will be impossible to distinguish it in such a way. In this case, when the lipid molecule in the inner or outer layer of the membrane is specified in the following sections of simulations and results, this suggests that the position of the lipid molecule is in the initial shape of the vesicle.

### 3.2. Simulation of Adding Lipid Molecules (1 in 50 Steps)

Simulations were performed by adding lipid molecules one by one to the following locations: (1) the inner membrane of the vesicle, (2) the outer membrane, and (3) the membranes on both sides. One lipid molecule is added every 50 steps, and this is performed up to 200,000 steps to double the number of lipid molecules.

#### 3.2.1. Adding to Inner Layer

**Figure 6:**
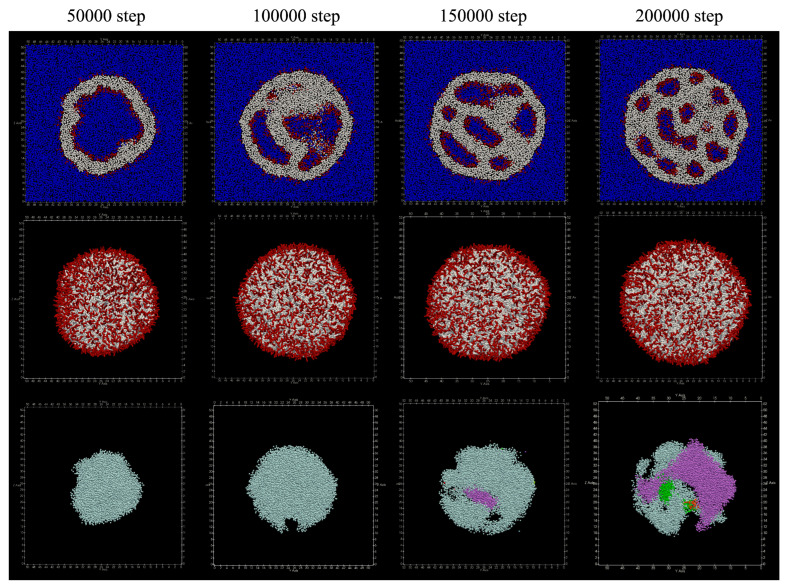
Shape changes when lipids are added to the inner membrane of the vesicle. Upper: cross section. Red represents hydrophilic group particles, white represents hydrophobic group particles, and blue represents water particles. Middle: appearance. Bottom: clustered water particles. Each color represents a separate cluster.

Structures obtained by adding lipids to the inner membrane of the vesicle are shown in Figure 6 From around 50,000 steps, the membrane inside the vesicle begins to protrude, and around 150,000 steps, the membrane is seen to stretch so as to divide the inside of the vesicle. Furthermore, it can be seen that the number of divided areas is increasing around 200,000 steps.

At 200,000 steps, the number of lipid molecules that moved from the inner membrane to the outer membrane was 16, and the number of lipid molecules that moved from the outer membrane to the inner membrane was five, so a total of 21 lipid molecules moved. No lipid molecules that migrated between the bilayer membranes were found. Table 2 shows the number of particles in the cluster of water particles. Since the data of No. 1 indicates the water particles located outside the vesicle, it can be seen that the number of clusters in the vesicle, that is, the number of compartments separated by the membrane, eventually increased to six.

#### 3.2.2. Adding to Outer Layer

**Figure 7:**
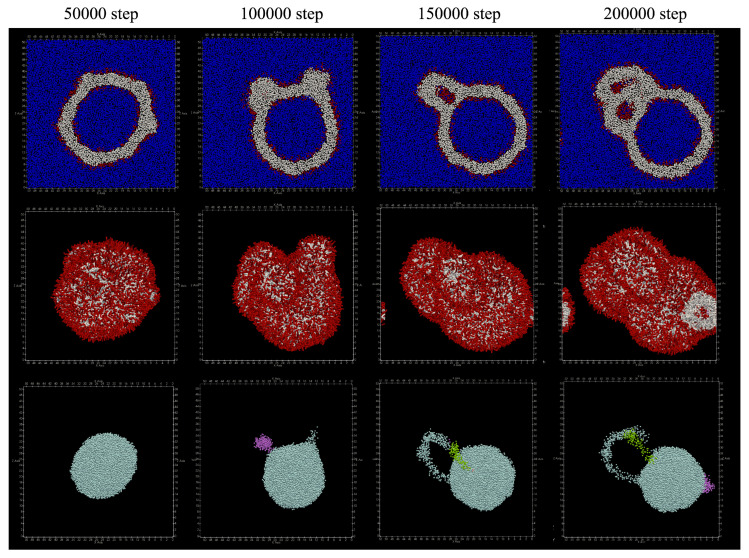
Shape changes when lipids are added to the outer membrane of the vesicle. Upper: cross section. Red represents hydrophilic group particles, white represents hydrophobic group particles, and blue represents water particles. Middle: appearance. Bottom: clustered water particles. Each color represents a separate cluster.

The structures obtained by adding lipids to the outer membrane of the vesicle are shown in Figure 7. At 100,000 steps, a structure was seen in which the outer membrane of the vesicle was projected. After that, at the 200,000 steps, it was observed that the protruding structure became a double membrane. In addition, no lipid molecules that migrated between the bilayer membranes were found. Table 3 shows the number of particles in the cluster of water particles. It can be seen that the number of clusters has finally increased to three or four.

#### 3.2.3. Adding Lipid Molecules to Both Layers

**Figure 8:**
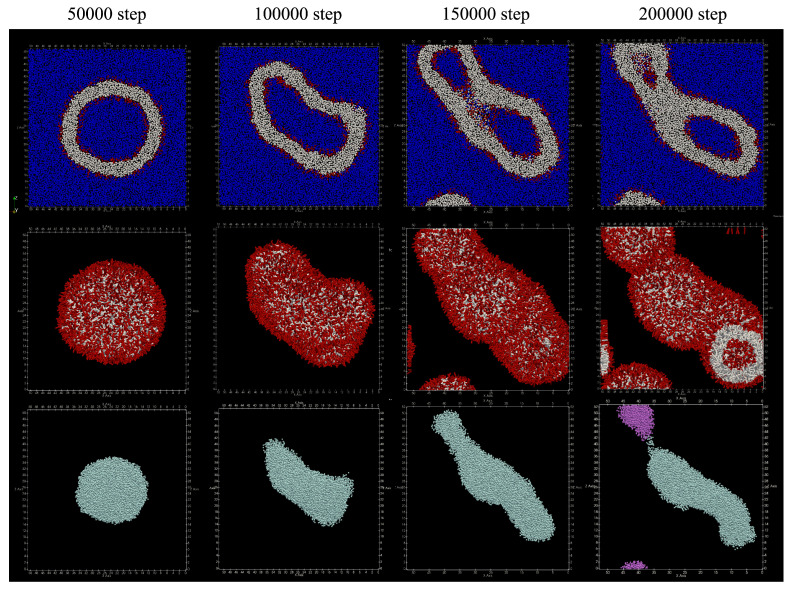
Shape changes when lipids are added to the membranes on both sides of the vesicle. Upper: cross section. Red represents hydrophilic group particles, white represents hydrophobic group particles, and blue represents water particles. Middle: appearance. Bottom: clustered water particles. Each color represents a separate cluster.

The number of lipid molecules added in the simulation was 1289 in the inner membrane and 2711 in the outer membrane. The number of lipid molecules that migrated from the inner membrane to the outer membrane was only three. The obtained structure is shown in Figure 8. Spherical vesicles were observed around 50,000 steps, and they were transformed into flat vesicles after the 100,000 steps. Around 200,000 steps, the water particles inside the vesicle are divided. From Table 4, the number of water particle regions inside the vesicle has increased to a few.

### 3.3. Shape Relaxation

We performed relaxation simulations of the shape of the vesicles, using the three types of structures obtained by the above simulations of adding lipid molecules every 50 steps as the initial structures. In the relaxation simulation, 200,000 steps were calculated under constant temperature and pressure conditions without adding lipid molecules.

**Figure 9:**
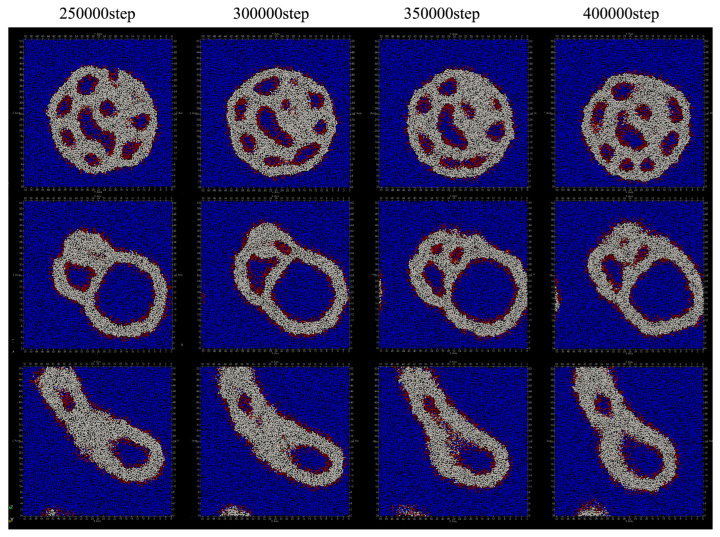
After stopping the addition of lipids to the vesicle, the structure was relaxed for 200,000 steps under constant temperature and pressure (200,000 to 400,000 steps): (**a**) lipid molecules have been added to the inner membrane of the vesicle; (**b**) lipid molecules have been added to the outer membrane of the vesicle; and (**c**) lipid molecules have been added to the membranes on both sides of the vesicle.

As a result of relaxing the structure obtained by adding lipid only to the inner membrane of the vesicle, there were 87 lipid molecules that migrated from the inner membrane of the bilayer to the outside, 20 that migrated from the outside to the inside, and a total of 107 lipid molecules that migrated between the bilayers. Figure 9a shows the relaxation process of the vesicle structure. The shape of the vesicle remained spherical during the simulation. Around 250,000 steps, membrane pores were formed in the vesicle, and water particles inside the vesicle were observed to move outward. The number of regions of water particles inside the vesicle divided by the membrane decreased from 11 to three.

On the other hand, as a result of relaxing the structure obtained by adding lipid only to the outer membrane of the vesicle, the number of lipid molecules that migrated from the inner membrane of the bilayer to the outside was three, and the number of lipid molecules that migrated from the outside to the inside was 87. Therefore, the total number of lipid molecules that migrated between the bilayers was 90. Figure 9b shows the vesicle shape relaxation process of the obtained structure. From the figure, it can be seen that the shape has not changed significantly through the simulation. The number of regions of water particles inside the vesicle divided by the membrane increased or decreased between four and six.

When the structure obtained by adding lipids to both layers of the vesicle membrane is relaxed, the number of lipid molecules that have moved outward from the inner membrane of the bilayer was six, and the number of lipid molecules that have moved from the outside to the inside was three. That is, there has been almost no movement of lipid molecules between the two layers of membranes. Additionally, as shown in Figure 9c, the shape of the vesicle has not changed significantly through the relaxation simulation. In addition, the three compartments inside the vesicle merged into one at around 250,000 steps and has not changed thereafter.

In the simulation of duplicating lipid molecules in the membrane and adding one lipid molecule every 50 steps, different vesicle shapes were obtained depending on the layer to which the lipid molecule was added. When a lipid molecule is added to the inner membrane of the vesicle, the inside of the vesicle is divided by the bilayer membrane, and when added to the outer membrane, the bilayer membrane protrudes to the outside of the vesicle. When lipid molecules were added to the membranes on both sides, the vesicles showed a flat structure. Furthermore, in the simulation to relax the obtained shape, a deformation was observed in which the number of regions of water particles in the divided vesicles was reduced.

### 3.4. Simulation of Adding Lipid Molecules (1 in 100 Steps)

By performing a simulation in which the frequency of addition of lipid molecules was halved, the deformation of the vesicle due to the difference in the dynamics of addition of lipids was investigated. In this simulation, once every 100 steps, one lipid molecule was duplicated and added into the bilayer, and 4000 lipid molecules were added to the vesicle membrane during 400,000 steps. As a result, the number of lipid molecules is twice the number in the initial state. The number of lipids in this final state is the same as in the simulation in which lipid molecules are added every 50 steps.

**Figure 10:**
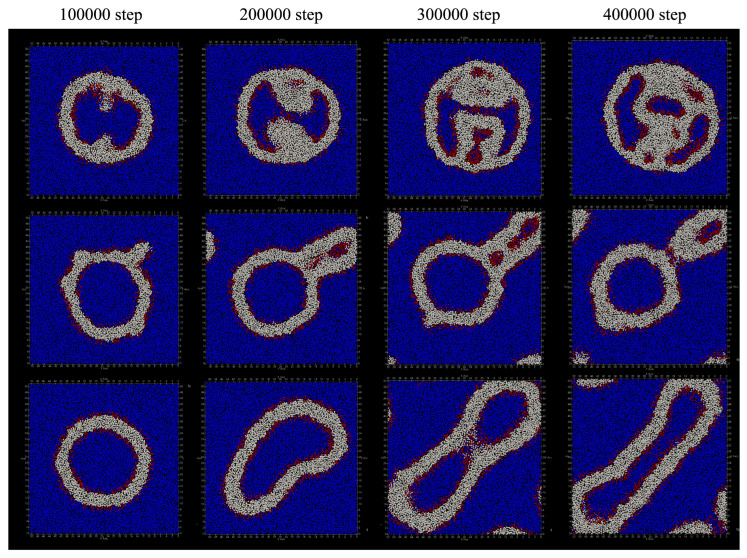
Change in vesicle shape when one lipid molecule is added to the vesicle membrane in every 100 steps (cross-sectional view). It was added by duplicating the lipid molecules of: (**a**) intima; (**b**) outer membrane; and (**c**) bilayer membrane of the vesicle.

The deformation process of the vesicle by adding lipid molecules every 100 steps is shown in Figure 10. Compared to the case where lipids are added every 50 steps, the vesicles are significantly deformed, but the characteristics of deformation due to changing the location where lipid molecules are added have not changed.

When added only to the inner membrane of the vesicle, the outer shape of the vesicle remains spherical, but a double membrane protrusion appeared from the inner membrane of the vesicle at 100,000 steps (Figure 10a). With the passage of time, the inside of the vesicle was divided into multiple regions by the protrusions of this membrane, and finally the number of divided regions became six. During the 400,000 steps, the number of lipid molecules that migrated from the inner membrane of the bilayer to the outside was 61, and the number of lipid molecules that migrated from the outside to the inside was 12. As the result, the total number of lipid molecules that migrated between the bilayers was 73.

**Figure 11:**
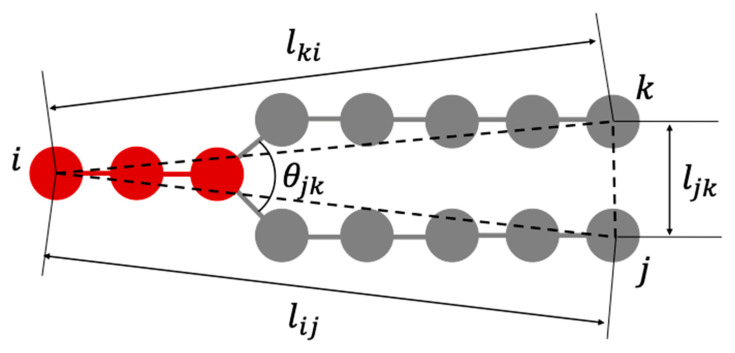
A model of a lipid molecule consisting of 13 DPD particles and parameters describing its shape. The particle at the end of the hydrophilic group is i, the particles at the ends of the two hydrophobic groups are j and k, and the distances between these particles are $l_{ki}$, $l_{ij}$ and $l_{jk}$. The angle formed by the bond between the two hydrophobic groups and the hydrophilic group is $\theta_{jk}$.

The deformation of the lipid bilayers constituting the vesicles is considered to be interdependent with the change in the steric structure of each lipid molecule. Therefore, we defined parameters that represent the shape of lipid molecules as shown in Figure 11, and investigated how these parameter values change during the deformation process of the vesicles shown in Figure 10. Although $l_{ij}$ and $l_{ki}$ are parameters that represent the length of lipid molecule, $l_{ij}$ is used when calculating statistics because of the symmetry of the molecule shape. The parameter $l_{jk}$ represents the distance between the ends of the two hydrophobic groups of the lipid molecule. $\theta_{jk}$ represents the angle formed by the roots of two hydrophobic groups.

**Figure 12:**
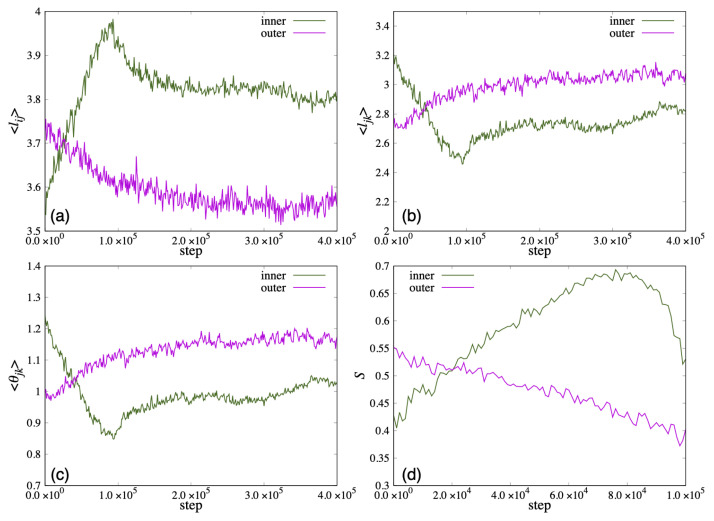
The change of the population average of microscopic parameters: (**a**) $l_{ij}$, (**b**) $l_{jk}$ and (**c**) $\theta_{jk}$ representing the shape of lipid molecules when lipid molecules are added to the inside of the membrane. (**d**) S is the order parameter of the director $u_{k}$ of the lipid molecules for the normal vector $n_{k}$ of the membrane surface of the vesicle.

In Figure 12, we show the time variation of the average length $\left\langle l_{ij}\right\rangle$ and the distance between the ends of the two hydrophobic groups $\left\langle l_{jk}\right\rangle$ that characterize the shape of the lipid molecule, and the average value of the angle formed by the roots of the two hydrophobic groups $\left\langle \theta_{jk}\right\rangle$. Because the vesicle deformed significantly from a spherical shape in 100,000 steps, the order parameter *S* could not be calculated accurately. Therefore, *S* was calculated up to 100,000 steps and plotted in (d). In addition, $\left\langle l_{ki}\right\rangle$ was omitted because $\left\langle l_{ki}\right\rangle$ was almost the same value as $\left\langle l_{ij}\right\rangle$. In early steps, the $\left\langle l_{ij}\right\rangle$ of the lipid molecule inside the bilayer membrane was shorter than the outside lipid. After 20,000 steps, the $\left\langle l_{ij}\right\rangle$ of the lipid molecule inside the bilayer membrane increased more than the outside lipid. Then, the $\left\langle l_{ij}\right\rangle$ increased, and the $\left\langle l_{jk}\right\rangle$, the angle $\left\langle \theta_{jk}\right\rangle$ decreased until 100,000 steps (Figure 12a–c). The order parameter *S* of the inner lipid increased until 80,000 steps and then decreased (Figure 12d).

On the other hand, while the $\left\langle l_{jk}\right\rangle$ and the angle $\left\langle \theta_{jk}\right\rangle$ of the lipid molecule in the outside of the bilayer decreased with time, the $\left\langle l_{ij}\right\rangle$ of the lipid molecule increased, shown in Figure 12a–c. The order parameter *S* of the outside lipid molecules decreased with time and the tilt angle of the lipid molecules increased.

When lipid molecules were added only to the outer membrane of the vesicle, the shape change of the vesicle up to 400,000 steps was observed as shown in Figure 10b. During this period, three lipid molecules moved outward from the inner membrane of the bilayer, and 54 molecules moved from the outside to the inside. At 100,000 steps, some double membrane protrusions were seen extending from the outer membrane of the vesicle. After 200,000 steps, two of the protrusions grew large, and multiple regions containing water particles were formed inside them. The area containing these water particles eventually increased to five.

**Figure 13:**
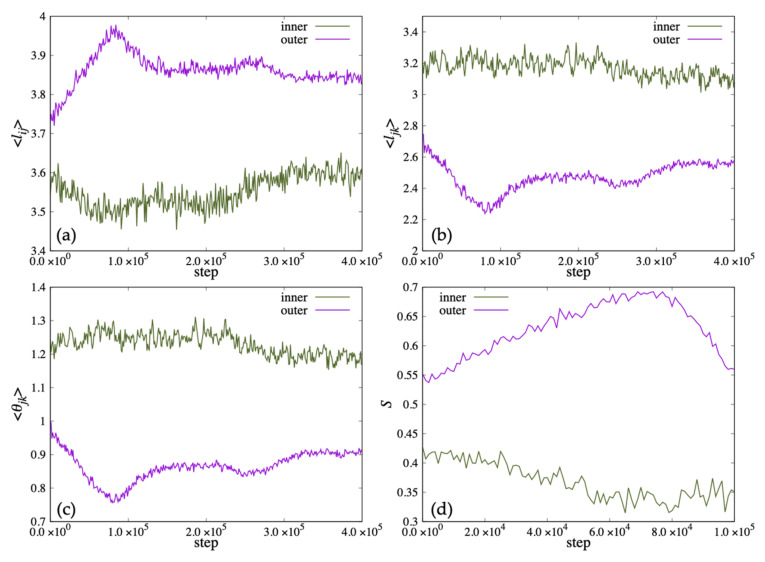
The change of the population average of microscopic parameters (**a**) $l_{ij}$, (**b**) $l_{jk}$ and (**c**) $\theta_{jk}$ representing the shape of lipid molecules when lipid molecules are added to the outside of the membrane. (**d**) S is the order parameter of the director $u_{k}$ of the lipid molecules for the normal vector $n_{k}$ of the membrane surface of the vesicle.

In Figure 13a–c, we show the time variation of the average length $\left\langle l_{ij}\right\rangle$, the average distance between the ends of the two hydrophobic groups $\left\langle l_{jk}\right\rangle$ and the average value of the angle $\left\langle \theta_{jk}\right\rangle$ for the lipid molecules inside the membrane. The lipid length $\left\langle l_{ij}\right\rangle$ of the inner lipid molecule decreased with time, and increased to almost the same value as the initial state after 300,000 steps. In addition, $\left\langle l_{jk}\right\rangle$ and $\left\langle \theta_{jk}\right\rangle$ decreased around the 220,000 steps. On the other hand, the outside lipid molecules increased to 80,000 steps and then decreased at 80,000 step and 260,000 step. In Figure 13d, the order parameter *S* of the lipid molecule inside decreased with time for the inner lipid molecule. The outside lipid molecule increased until 80,000 steps and then decreased.

Figure 10c shows the structural change of the vesicle when lipid molecules are added to both the inside and outside of the membrane. During this period, the only lipid molecule that moved between the bilayer membranes was one lipid molecule that moved outward from the inner membrane of the bilayer membrane. The vesicle remained spherical up to 100,000 steps, but deformed flat at 200,000 steps. After 300,000 steps, the shape of the vesicle was deformed to extend like a tube. Additionally, unlike the case where lipid molecules were added to the outside or inside of the membrane, the inside of the vesicle was not divided by the membrane.

**Figure 14:**
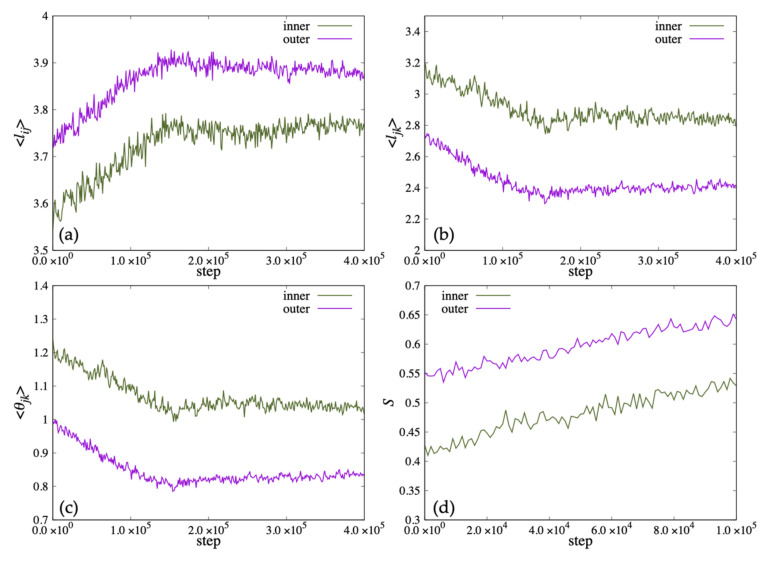
The change of the population average of microscopic parameters (**a**) $l_{ij}$, (**b**) $l_{jk}$ and (**c**) $\theta_{jk}$ representing the shape of lipid molecules when lipid molecules are added to the both sides of the membrane. (**d**) S is the order parameter of the director $u_{k}$ of the lipid molecules for the normal vector $n_{k}$ of the membrane surface of the vesicle.

The time change of the average length $\left\langle l_{ij}\right\rangle$, $\left\langle l_{jk}\right\rangle$ and the average angle $\left\langle \theta_{jk}\right\rangle$ for the lipid molecules are shown in Figure 14. The average length $\left\langle l_{ij}\right\rangle$ of lipid molecules increased to 150,000 steps and then became constant for both inside and outside the bilayer. On the other hand, $\left\langle l_{jk}\right\rangle$ and the average angle $\left\langle \theta_{jk}\right\rangle$ decreased until 150,000 steps and then became constant. The order parameter *S* increased for both layers of lipid, and the tilt angle decreased with time.

## 4. Discussion

From the simulation results in the previous section, it was found that the deformation process of vesicles changes depending on the position of lipid molecules incorporated into the lipid bilayer. These deformation processes resemble the shape change during the division of L-form cells, as shown in Figure 1. Several protrusions were created on the outside of the vesicle when lipid molecules were added to the outer layer of the vesicle or on the inside of the vesicle when added to the inner layer. As the protrusions grow, semi-vesicle-like structures [12] are formed inside or outside the vesicles causing topological changes such as membrane division. The shapes obtained by adding lipid molecules to outside membrane look like the “budding” observed during the division process of L-form cells [1,2]. On the other hand, the shape was similar to multi-vesicular endosome (MVE) [24] when lipid molecules were added inside. When lipid molecules were added to both sides of the vesicle membrane, the shape of the entire vesicle was deformed so as to extend like a tube. This seems to be a shape change corresponding to the “tubulation” observed during the division process of L-form cells [2]. In addition, in the simulation that relaxes the structure obtained by the simulation of adding lipid molecules, deformations such as returning to the vesicle of a single membrane and forming a structure close to the vesicle on the outside were observed.

Considering the relationship between the deformation of the vesicle and the behavior of the lipid molecule from a microscopic point of view, we focus on the shape of lipid molecules and their movement in the lipid bilayer, especially the flip-flop. The frequency of flip-flops of lipid molecules in the membrane was higher when lipid molecules were added to only one layer than when they were added to both layers of the membrane. Since the molecular density in the layer where the lipid molecule was added became high, the lipid molecule moved to the other layer in which the lipid molecule was not added. In addition, when lipid molecules were added to the inner layer of the membrane, protrusions of the lipid bilayer membrane were generated on the inner membrane of the vesicles, and when lipid molecules were added to the outer layer, membrane protrusions were formed on the outer membrane. That is, the excess lipid molecules in one layer move to the other layer by flip-flop, reducing the free energy due to strain between lipid molecules. However, if lipid molecules are added before the free energy due to the strain is sufficiently relaxed, the free energy of local strain in the layer to which the lipid molecules are added increases, and as a result, protrusions on the outside of the bilayer membrane will form as a new membrane structure. These bilayer protrusions are thought to have a structure similar to BP, found by Nakagawa et al. [13].

In other words, the addition of lipid molecules increases the potential energy locally, and the kinetic energy of the particles that make up the lipid molecules increases accordingly. As a result, the lipid molecules that make up the membrane may have strong repulsion with each other, resulting in a structure in which the bilayer membrane protrudes. Similar to the study by Baoukina et al. [12], the further addition of lipid molecules may cause the bilayer projections to bend and deform to form vesicles.

In the simulation, in which lipid molecules were added to both layers of the membrane, the shape of the vesicle was observed to change in an elongated manner. The frequency of flip-flopped lipid molecules was smaller than that of the simulation added to one layer of the membrane. The lipid molecular densities of the outer layer and the inner layer of the membrane may increase evenly, so that the area of both layers increased while suppressing the increase of strain due to the area difference elasticity between the two layers. As a result, the vesicle was deformed instead of the membrane protrusions observed in the simulation added to one side of the layer.

**Figure 15:**
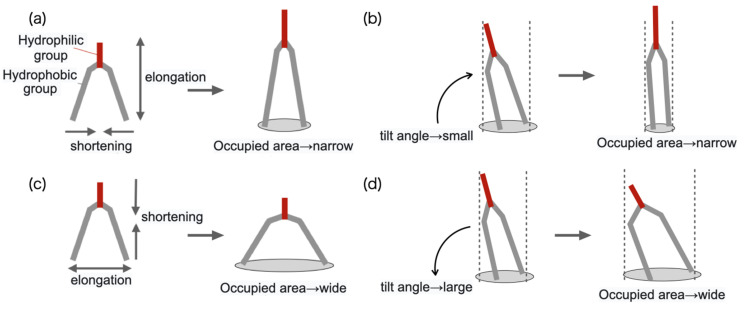
Changes in the shape of lipid molecules: (**a**) lipid molecules extend perpendicular to the membrane surface; (**b**) lipid molecules extend toward the membrane surface; (**c**) lipid molecules decrease tilt angle; and (**d**) lipid molecules increase tilt angle.

In addition, $l_{ij}$ of lipid molecules constituting the layer to which lipid molecules were added tended to increase, and $l_{jk}$ tended to decrease as shown in Figure 15a. The shape of the lipid molecule may have been deformed so as to elongate to lipid direction. Moreover, the order parameter *S* was increasing, which implies that the tilt angle was decreasing, as shown in Figure 15b. These are because the area of the membrane that can be occupied by one lipid molecule has decreased due to the addition of the lipid molecule. On the other hand, the $l_{ij}$ of the lipid molecules constituting the layer to which the lipid molecule was not added decreased, and the $l_{jk}$ tended to increase. In other words, the shape of the lipid molecule was deformed so as to extend laterally toward the membrane surface as shown in Figure 15c. Furthermore, the order parameter *S* decreased and the tilt angle increased as shown in Figure 15d. Since the area of the layer to which the lipid molecule was added increased, the area of the paired layer increased accordingly, and the deformation that expanded the occupied area of each lipid molecule occurred. This corresponds to the deformation to reduce the area difference elastic energy between the layers of the membrane.

During the deformation of the vesicle, the length $l_{ij}$ of the lipid molecules constituting the layer to which the lipid molecules were added tended to return to the original length. It is supposed that the individual lipid molecules were deformed to their original stable shape due to the change in the global shape.

In the simulation of structural relaxation of a vesicle with multiple regions separated by a membrane, the final structure obtained by the simulation, in which lipid molecules were added every 50 steps, was used as the initial structure. When the structure obtained by the simulation of adding lipid molecules to the inner layer or both layers of the membrane was used as the initial structure, the number of water regions inside the vesicle divided by the membrane decreased with time. The shape observed during the addition of lipid molecules is thought to be the characteristic structure found in the non-equilibrium state. Therefore, it is expected that the vesicle will eventually return to a more stable single-membrane vesicle.

In the relaxation simulation of the structure, generated by adding lipid molecules to the outer membrane, the number of regions divided by the bilayer membrane repeatedly increased and decreased, as shown in Figure 9b. This indicates that on the outside of the vesicle, the semi-vesicle structures [12], which were formed by bending and fusing the protrusions of the bilayer membrane, repeats generation and disappearance in thermal fluctuation. This may indicate that the free energy barrier for returning to the single membrane vesicle is high. Therefore, even if the relaxation simulation is continued for a longer period of time, it is possible that the shape will be different from that of the single bilayer vesicle, causing vesicular division through budding.

Table 5 shows the number of lipid molecules flip-flopped per 10,000 steps in the addition and relaxation simulations.

The frequency of flip-flops per unit time was higher when lipid molecules were added every 100 steps than when lipid molecules were added to one layer every 50 steps. Flip-flops are generated to eliminate the strain generated in the membrane structure; the longer the time interval for adding lipid molecules, the more efficiently the strain in the membrane structure can be eliminated. In addition, in the simulation that relaxes the structure obtained by adding lipid molecules every 50 steps, more lipid molecules flip-flopped per unit time than in the simulation that added every 100 steps. The lipid molecules that could not be flip-flopped in the additional simulation flip-flopped during relaxation.

On the other hand, in the simulation in which lipid molecules were added to both layers, there were fewer lipid molecules that flip-flop than those in which lipid molecules were added to one side, including the case of relaxation. This is a result of suppressing the increase in free energy due to the area difference between the two layers by adding lipid molecules proportional to the number of lipid molecules in both layers.

## 5. Conclusions

In this study, we performed a simulation in which lipid molecules were added to the lipid bilayer vesicle and then relaxed. Additions to the outer layer and both layers resulted in a shape similar to an L-form cell. On the other hand, additions to the inner layer did not obtain that shape. In addition, in the simulation that relaxes the structure obtained by the simulation of adding lipid molecules, deformations such as returning to the vesicle of a single membrane and forming a structure close to the vesicle on the outside, were observed.

Though we were able to observe the shape change accompanied by the topology change as mentioned above, we could not reproduce the division of vesicle. In order to confirm the state of division, it may be necessary to perform a longer simulation and to make the interval of adding lipid molecules slower.

Due to computer specifications, in this study, simulations were performed using a small vesicle with a radius of $15r_{c}=10.5$ nm as a model. Adding lipid molecules directly to the membrane also shortened the calculation time. In order to calculate with a larger system, it is necessary to devise ways to reduce the calculation time. Simulations of larger vesicles using some useful technique may show shape changes in more metastable states seen during the division of L-form cells, as well as the MVE [24] observed in vivo.

The important points suggested by the simulation results in this study are as follows. It was shown that when lipids were added to the outer layer of the lipid bilayer that forms the vesicle, the area of the outer layer increased and the vesicle grew via the budding path. It was also found that when lipids were added to both layers of the lipid bilayer, the vesicles grew elongated and deformed like tubulation. These closely resemble the deformations seen in the growth of L-form cells. On the other hand, when lipids were added to the inner layer of the lipid bilayer, the interior of the vesicle was divided into several regions separated by membranes. Although this morphology has not been observed in the growth of L-form cells, similar morphology has been observed in MVE. Thus, it is expected that the pathway of shape change during division of L-form cells is selected depending on which layer of the cell membrane the lipid is added to.

## Figures and Tables

**Table 1:** Value for $a_{ij}$ for each particle type.

$a_{ij}$	Water	Hydrophilic	Hydrophobic
water	25	25	100
hydrophilic	25	25	100
hydrophobic	100	100	25

**Table 2:** The number of water particles that make up each cluster when a lipid molecule is added to the inner layer of the vesicle.

Cluster Number	Step			
	50,000	100,000	150,000	200,000
cluster1	331,654	332,124	332,019	331,490
cluster2	20,089	25,646	24,805	11,302
cluster3			1603	9759
cluster4			20	369
cluster5			28	1260
cluster6			12	116
cluster7			1	34

**Table 3:** The number of water particles that make up each cluster when a lipid molecule is added to the outer layer of the vesicle.

Cluster Number	Step			
	50,000	100,000	150,000	200,000
cluster1	337,048	341,643	346,035	331,490
cluster2	15,543	16,233	17,646	18,256
cluster3		691	28	1338
cluster4			531	714
cluster5			35	

**Table 4:** The number of water particles that make up each cluster when a lipid molecule is added to the both layers of the vesicle.

Cluster Number	Step			
	50,000	100,000	150,000	200,000
cluster1	335,465	339,281	343,337	347,378
cluster2	17,311	19,103	20,925	19,594
cluster3	1			2971
cluster4				714

**Table 5:** Number of lipid molecules flip-flopped per 10,000 steps.

	Number of Lipid Molecules Transferred per 10,000 Steps		
	From Inside to Outside	From Inside to Outside	Total
**Addition (1 in 50 steps)**			
**inner layer**	0.8	0.25	1.05
**outer layer**	0	0	0
**both layer**	0.15	0	0.15
**Addition (1 in 100 steps)**			
**inner layer**	1.525	0.3	1.825
**outer layer**	0.075	1.35	1.425
**both layer**	0.025	0	0.025
**Relaxation**			
**inner layer**	2.175	0.5	2.675
**outer layer**	0.575	2.175	2.25
**both layer**	0.15	0.075	0.225

## Data Availability

Data is contained within the article.
